# Wear Behaviours and Oxidation Effects on Different UHMWPE Acetabular Cups Using a Hip Joint Simulator

**DOI:** 10.3390/ma11030433

**Published:** 2018-03-16

**Authors:** Saverio Affatato, Alessandro Ruggiero, Sami Abdel Jaber, Massimiliano Merola, Pierangiola Bracco

**Affiliations:** 1 Medical Technology Laboratory, IRCCS—Rizzoli Orthopaedic Institute, Via di Barbiano, 1/10, 40136 Bologna, Italy; jaber@tecno.ior.it; 2 Department of Industrial Engineering, University of Salerno, 84084 Fisciano, Italy; ruggiero@unisa.it (A.R.); mmerola@unisa.it (M.M.); 3 Chemistry Department and Nanostructured Interfaces and Surfaces (NIS) Centre, University of Turin, Via Giuria 7, 10125 Turin, Italy; pierangiola.bracco@unito.it

**Keywords:** vitamin-E stabilized PE, cross-linked PE, standard PE, hip simulator, FTIR analysis

## Abstract

Given the long-term problem of polyethylene wear, medical interest in the new improved cross-linked polyethylene (XLPE), with or without the adding of vitamin E, has risen. The main aim of this study is to gain further insights into the mutual effects of radiation cross-linking and addition of vitamin E on the wear performance of ultra-high-molecular-weight polyethylene (UHMWPE). We tested four different batches of polyethylene (namely, a standard one, a vitamin E-stabilized, and two cross-linked) in a hip joint simulator for five million cycles where bovine calf serum was used as lubricant. The acetabular cups were then analyzed using a confocal profilometer to characterize the surface topography. Moreover; the cups were analyzed by using Fourier Transformed Infrared Spectroscopy and Differential Scanning Calorimetry in order to assess the chemical characteristics of the pristine materials. Comparing the different cups’ configuration, mass loss was found to be higher for standard polyethylene than for the other combinations. Mass loss negatively correlated to the cross-link density of the polyethylenes. None of the tested formulations showed evidence of oxidative degradation. We found no correlation between roughness parameters and wear. Furthermore, we found significantly differences in the wear behavior of all the acetabular cups. XLPEs exhibited lower weight loss, which has potential for reduced wear and decreased osteolysis. However, surface topography revealed smoother surfaces of the standard and vitamin E stabilized polyethylene than on the cross-linked samples. This observation suggests incipient crack generations on the rough and scratched surfaces of the cross-linked polyethylene liners.

## 1. Introduction

Ultra-high-molecular-weight polyethylene (UHMWPE) is a particular type of polyethylene (PE) with an exceptionally high molecular mass. It is a unique polymer with outstanding properties, in terms of chemical inertness, lubricity, impact, and abrasion resistance. Despite coming from a family of polymers with an extremely simple chemical composition, consisting of only hydrogen and carbon, UHMWPE shows a complex hierarchy of organizational structures at the molecular and supermolecular length scales [1]. Besides the molecular mass, the microstructure of the polymer also plays an important role in determining its physical, chemical, and mechanical properties. UHMWPE, as most polyethylenes, is a semi-crystalline polymer, composed of at least two interpenetrating phases: a crystalline, ordered phase, and an amorphous disordered one, possibly intercalated by a partially ordered interphase. Such a precise combination of chemical structure, molecular mass, and microstructure is at the basis of the peculiar balance of high mechanical properties and wear resistance that has made UHMWPE the material of choice in arthroplasty [2].

Thanks to its unique properties, UHMWPE is the most used material in hip joint replacement, being the soft insert coupled with harder materials (typically ceramics and metals). Such coupling shows very good tribological performances in terms of friction and wear [3,4]. Unfortunately, oxidative degradation can decrease its mechanical properties, leading to debris production and eventual osteolysis and implant loosening [5,6,7]. It is believed that wear of UHMWPE is to take place via plastic deformation of the polymer, with molecular alignment in the direction of motion that results in the formation of fine, drawn-out fibrils oriented parallel to each other. As a result of this arrangement, the UHMWPE wear surface may strengthen along the direction of sliding, while it weakens in the transverse direction [2]. Many efforts were done to improve the mechanical and molecular characteristic of the UHMWPE, as by cross-linking its molecular chains or by doping it with vitamin E [8]. Radiation cross-linking was demonstrated to improve the wear resistance of UHMWPE. On the other hand, irradiated polyethylene has also shown an unacceptably low oxidation stability [2]. As a consequence, stabilization strategies were developed in order to minimize post-irradiation oxidative ageing. Basically, two different strategies were adopted: one involved a thermal treatment of the polyethylene (re-melting or annealing), while the other included the addition of an anti-oxidant stabilizer. Oral and co-workers [9], suggested that the re-melting of the polyethylene reduces crystallinity and fatigue properties. Therefore, vitamin E was introduced to solve the oxidation problem [10]. Some authors [11,12,13], suggest to incorporate vitamin E in UHMWPE through blending the vitamin in the UHMWPE powder and then cross-link the blend through irradiation. With this process, the presence of vitamin E should protect the radiation-cross-linked polymer from oxidation, thus avoiding re-melting.

Preclinical evaluation of new biomaterials is necessary, and it could be considered as an extension of the risk analysis [14,15]. The wear performance of these improved biomaterials is often evaluated using hip joint simulators. Hip wear simulation tests are used since 40 years ago, and they represent a powerful system to assess the improvement in wear resistance before clinical use [16,17]. Some authors [18,19,20] observed a reduction in wear rate by the addition of vitamin E to highly cross-linked UHMWPE compared to conventional UHMWPE. However, there is a trade-off in determining the oxidation stability and the reduction in wear rate between the radiation dose applied for cross-linking and the amount of vitamin E incorporated into the polymer. Affatato and co-workers [21], using a hip joint simulator, found that the cross-linked polyethylene (XLPE) blended with vitamin E wore more than XLPE and conventional UHMWPE.

Each potential innovation has been accompanied by a great deal of pre-clinical trials, performed by researchers all over the world, often with very different methods and sometimes with contradictory results. In this regard, to go more in depth in the wear and oxidation behavior of new formulations of UHMWPE, we asked whether the addition of vitamin E on conventional UHMWPE could improve its wear performance in comparison with highly cross-linked polyethylene.

## 2. Materials and Methods

### 2.1. Specimens Tested

Four different batches of UHMWPE acetabular cups (32 mm inner × 50 mm outer dimensions; 6 specimens for each batch) coupled with 32 mm cobalt–chromium–molybdenum (CoCrMo) femoral heads have been investigated using a hip joint simulator. Three components of each batch run onto the simulator following a standardized procedure [22], another three acetabular cups for each type of material used were stored (non-loaded) in bovine calf serum to compensate for weight changes due to fluid absorption. All polyethylenes tested in this study were machined from polymer bars Chirulen GUR 1020 (Polymax, Adler, Milan, Italy). Cross-linked acetabular cups were firstly γ-ray irradiated with 50 and 75 kGy (±10%), then thermally treated at 150 °C (re-melted), in order to remove free radicals formed during irradiation (hereinafter called XL-50 and XL-75). After these treatments, the cups were machined to their final shape. Similarly, vitamin E-containing (0.1% mass), UHMWPE acetabular cups (hereinafter called VE) were machined from a vitamin E-blended UHMWPE bars (Polymax, Adler, Milan, Italy). The UHMWPE that not received any treatment were called standard PE (STD). All the cups were then subjected to ethylene oxide sterilization (ETO). All polyethylene acetabular cups were pre-soaked for four weeks in a bath of deionized water prior the wear tests.

### 2.2. Experimental Wear Details

Wear test was performed using a 12-station hip joint simulator (IORSynthe, Bologna, Italy) [9]. The test was carried out applying the kinematic inputs and outputs as recommended by ISO 14242-1:2012. The simulator utilizes hydraulic actuators to apply the cyclic vertical compressive loads (oscillating between 300 and 3000 N). The lubricant used was 25 vol % newborn calf serum balanced with distilled water, with 0.2% (mass) sodium azide in order to retard bacterial growth, and 20 mM EDTA (ethylenediaminetetraacetic acid) to minimize precipitation of calcium phosphate. The mass loss of the cups was determined every 0.5 million cycles (Mc) using a microbalance (Sartorius Cubis Mse 225 S-000-DU, Goettingën, Germany) with a resolution of 0.01 mg and an uncertainty of 0.01 mg. Before the weighting operation, the specimens were cleaned from dust and possible debris using a dedicated detergent (Clean 70, Elma GmbH, Düsseldorf, Germany) in an ultrasonic bath maintained at 40 °C for 10 min. After rinsing, the cups were put back in the ultrasonic bath with deionized water for an additional 15 min. The cups were then dried with nitrogen gas. During any interruption of the test (every 500 × 10^5^ cycles), the cups were stored in a closed, dust-free container at 70% of relative humidity. The test was re-started with fresh serum solution.

The test lasted five Mc, as recommended by the international guidelines [23], under environmental temperature conditions. The wear trend was determined from the mass loss of each acetabular cup, corrected by acetabular soak control. The wear rates, calculated from the steady-state slopes of the mass loss versus number of cycles, were obtained using least squares linear regression. The mass loss data were analyzed using a nonparametric Kruskall–Wallis (K–W) test; statistical significance was set at *p* < 0.05.

### 2.3. Surface Topography Characterization

**Figure 1:**
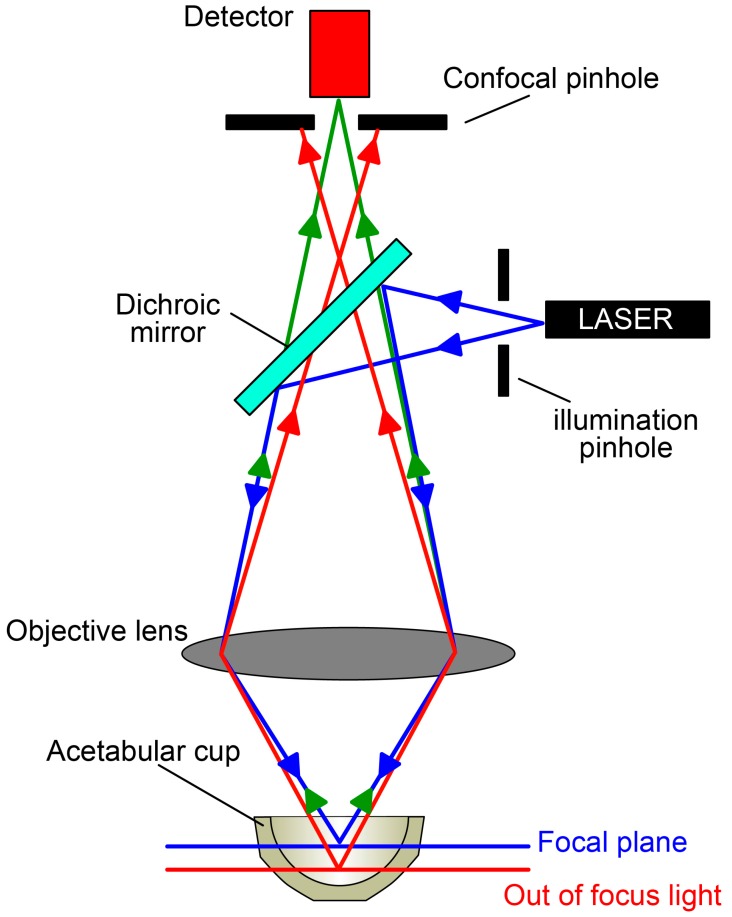
Schematic representation of the confocal apparatus for the topography acquisitions.

The topographic analyses were performed using a PLu Neox profilometer (Sensofar, Terrassa, Spain), capable to gain three-dimensional images of a surface, operating either as confocal microscope or as white light interferometer with a vertical resolution, declared by the manufacturer, of less than 0.1 nm. In this study, we selected the confocal lens of 20× magnifications, while the acquisition lengths were adjusted to compensate the inner curvature of the surfaces. Such a non-contact instrument was selected to measure the roughness of the polyethylene cups as optical techniques do not risk damaging the surface under investigation. On the other hand, a traditional roughness profilometer can scratch the surface of a soft material, such as the UHMWPE. The acquisition process followed an established procedure [24], where the polyethylene liners were first cleaned from debris, as described for the wear measurement, and—right before each acquisition—cleaned with ethanol and allowed to dry under a controlled environment in ambient air. In Figure 1, a schematic representation of the acquisition apparatus is shown.

The topography characterization was realized on eight selected UHMWPE acetabular cups. In particular, we analyzed the most worn acetabular cups for each configuration, plus the corresponding check-control, to compare worn and unworn acetabular cups. The topographical acquisitions were realized to gain qualitative information on the surface condition of the samples after five Mc of test running. Furthermore, it was obtained a quantitative analysis in term of surface roughness. To do so, a Gaussian filter was applied according to the international guidelines in ISO 4287:1997 [25]. A cut-off wavelength of 80 μm was selected along with an evaluation length of 400 μm—equivalent to 5 times the cut-off. The roughness parameters selected were Ra, Rq, Rz, and Rt. Ra defines the absolute of the mean deviation of the irregularities from the mean line, over a sampling length. Rq is the standard deviation of the distribution of surface heights. Rz is the difference in height between the average of the five highest peaks and the five deepest valleys. Rt is the maximum height of the profile, defined as the vertical distance between the highest peak and the lowest valley along the measurement length.

### 2.4. FTIR Spectroscopy

All four different biomaterials were characterized by means of a Fourier Transformed Infrared Spectroscopy (FTIR) Microscope (Spectrum Spotlight 300, Perkin-Elmer, Shelton, CT, USA). A series of 180 μm thick slices was obtained from the cups cross section, using a PolyCuts Microtome (Leica Microsystem, Wetzlar, Germany) at 10 mm/s in air at room temperature. Line-scan spectra were collected on a 100 × 100 μm^2^ area (resolution 4 cm^−1^, 16 scans per spectrum), every 100 μm along the mapping direction, starting from the articulating surface towards the bulk. All spectra were normalized at 2020 cm^−1^ at an absorption of 0.05, corresponding to a film thickness of ca. 100 µm. The combination band at 2020 cm^−1^, associated with the twisting of CH_2_, was used as an internal standard, since it can be regarded as unaffected by minor changes in the polymer structure. The molar concentration of trans-vinylene double bonds was calculated from the 965 cm^−1^ absorption bands, using the well-established molar absorptivity proposed by De Kock and Hol [26].

### 2.5. Determination of Cross-Link Density and Crystallinity

The cross-link density of each sample was quantified by gravimetric swelling. Small cylinders with diameter of 5 mm and approximate weight of 15 mg were cut from the control cups and immersed in 25 mL of xylene at 135 °C for 3 h to reach the equilibrium swelling. The initial weight and xylene uptake were used to calculate the swell ratio and the cross-link density, using a validated protocol [21].

The crystallinity of the test samples was determined using a differential scanning calorimetry (DSC 6-Perkin-Elmer, Waltham, MA, USA) at a heating rate of 10 °C/min. The sample weights varied around 5 mg. The heat of fusion was calculated by integrating the DSC endotherm from 60 to 160 °C. The crystallinity was calculated by normalizing the heat of fusion to the heat of fusion of 100% crystalline polyethylene (293 J/g) [27].

## 3. Results

**Figure 2:**
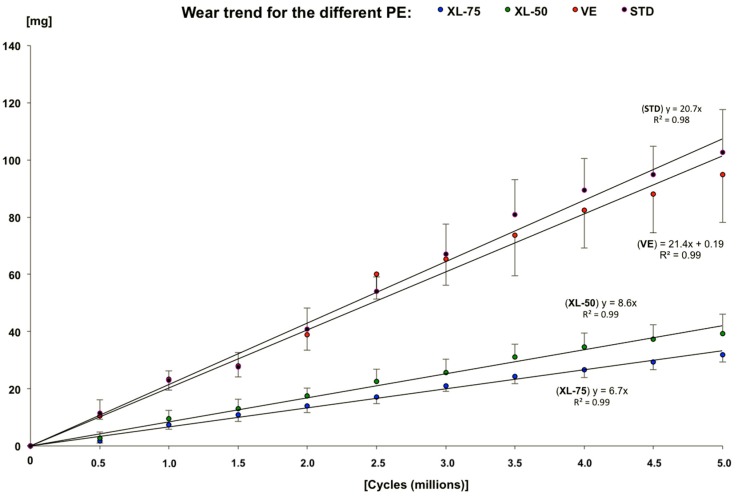
Wear behavior for the different configurations of polyethylene tested.

All the polyethylene acetabular cups completed the planned five Mc. As showed in Figure 2, where the different polyethylene liners are compared, a higher mass loss rate for the standard polyethylene (STD) than for the other combinations was found. Close to this trend, the VE cups have a slightly smaller wear rate.

The polyethylene named XL-75 maintained the lowest mass loss than the other configurations during the whole test, as confirmed by the results of the post hoc test (Table 1). Statistical significant differences were observed between XL-75 vs UHMWPE and VE (*p* = 0.015 and *p* = 0.02, respectively). No statistically significant differences (*p* > 0.05) were observed between the other configurations of polyethylene cups using the K-S statistical test (Table 1).

### 3.1. Surface Topography Analysis

**Figure 3:**
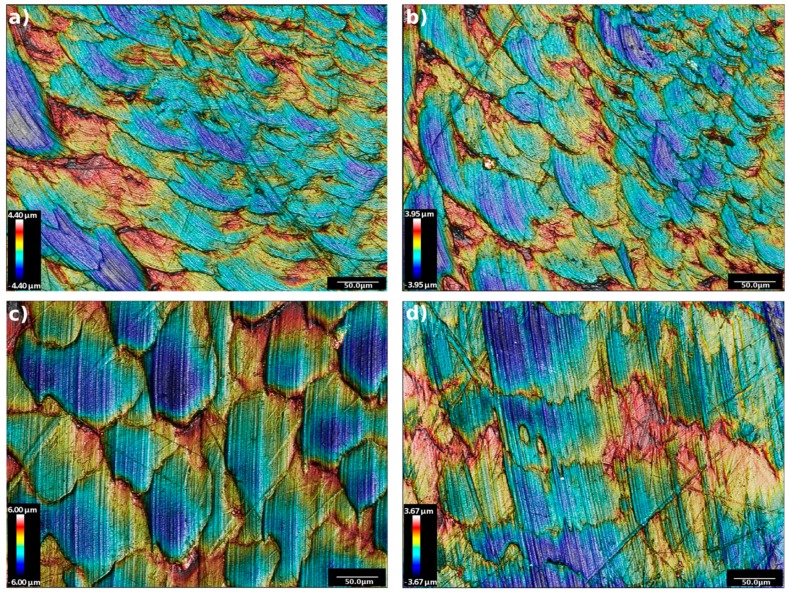
Contour images of the topographies acquired on the inner surfaces of four non-loaded specimens: (**a**) VE; (**b**) STD; (**c**) XL-75; (**d**) XL-50.

**Figure 4:**
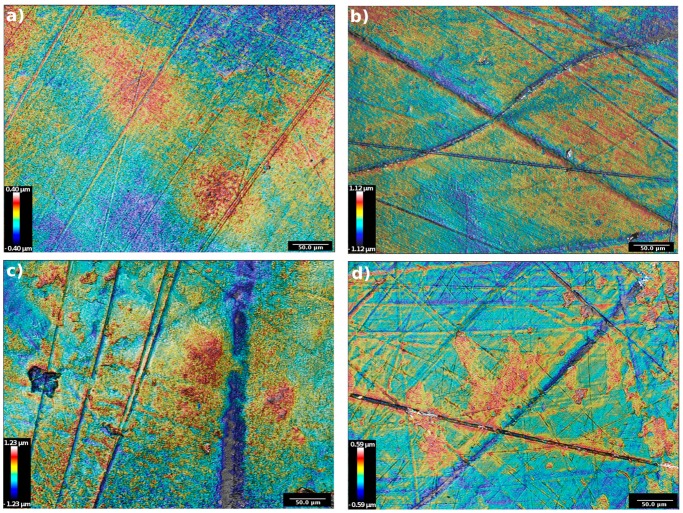
First set of contour images of the topographies acquired on the worn inner surfaces of four loaded specimens: (**a**) VE; (**b**) STD; (**c**) XL-75; (**d**) XL-50.

Surface topography of the samples is shown in Figure 3 and Figure 4, where contour images for each analyzed cup are presented. The different range of the height scales is influenced by the inner curvature of the cups. In Figure 3, the image shows the inner surfaces of the soak cups, which did not undergo wear simulation. The contours images of the topography present a similar pattern, typical of a clear UHMWPE surface for hip implants. In fact, the polymer has a lamellar shape, characterized by fine scratches, deriving from the polishing phase. Nevertheless, few long and transversal scratches are visible all over the images.

In Figure 4 are presented the contour topographies of the worn cups, after five million cycles of wear test. The images highlight that the inner surface of the cups is mainly characterized by the presence of grooves and scratches. In Figure 4a, a VE cup presents an almost smooth surface crossed by long and fine scratches; no signs of depth wear nor delamination are visible. In Figure 4b the inner surface of an STD cup is characterized by more frequent and larger grooves than on the VE. These paths are crossed in different directions. Also, signs of delamination are found along the border of these scratches. Figure 4c presents a worn area relative to a XL-75 cup. In this case, rough scratches are visible along with a deep groove (on the right side) and a worn crater (left side). In Figure 4d is shown a worn surface of a XL-50 cup, the surface presents many scratches in multiple directions and recurrent signs of wear along the side of these lines. Further worn images on the different configurations of the polyethylene tested, are shown in the supplementary section (Figure S1).

**Figure 5:**
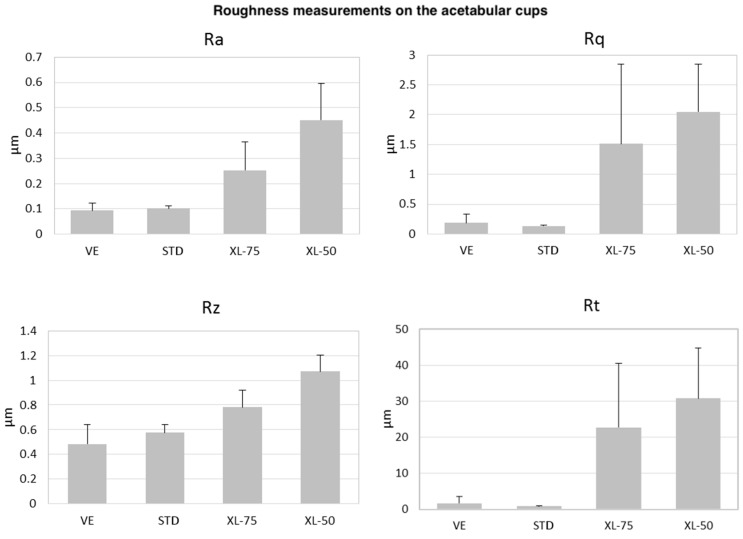
Roughness histograms of the worn surfaces on the mentioned cups. The parameters are Ra, Rq, Rz, and Rt.

These qualitative analyses were combined with roughness measurements that are summarized in Figure 5. Ra values are equal to 0.09, 0.10, 0.25, and 0.45 μm respectively for the VE, STD, XL-75 and XL-50 cups. Rq is 0.19, 0.13, 1.5, and 2.1 μm, respectively, for VE, STD, XL-75, and XL-50; these values highlight how the deviation from the mean line is higher on the rougher surfaces of the cross-linked polyethylene. Rz and Rt provide evidence of the presence of elevate peaks and deep valleys along the measured profiles, and our analysis shows considerably higher values for the cross-linked polyethylene than the standard and the vitaminized ones. The values of Rz are 0.48, 0.57, 0.78, and 1.07 μm, respectively, for VE, STD, XL-75 and XL-50, whereas for the Rt parameters these are 1.69, 0.85, 22.7, and 30.7 μm, in the same order of correspondence.

### 3.2. FTIR Spectroscopy and Cross-Link Density Results

The physical–chemical characteristics of the control samples, resulting from FTIR, DSC, and cross-link density measurements, are summarized in Table 2.

**Figure 6:**
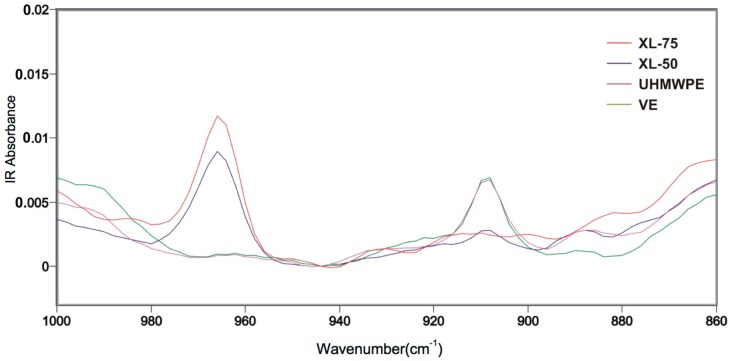
FTIR spectra of the investigated materials.

Figure 6 shows the FTIR spectra of all materials. The VE sample spectrum does not show any significant difference from that of virgin UHMWPE (STD). On the contrary, the spectra of the irradiated samples show a decrease in the vinyl double bonds absorption at 909 cm^−1^ and the appearance of an additional absorption at 965 cm^−1^, attributed to the formation of trans-vinylene double bonds, whose concentration is constant along the cup section, and increases with the radiation dose (see Table 2). No traces of oxidation products in the 1700 cm^−1^ area were observed in any of the samples (not shown). No significant differences in crystallinity were found between the STD and VE samples, while a significant decrease was observed in the crystallinity of the cross-linked polyethylenes. The cross-link density (ν_d_) was measurable on the irradiated samples only (XL-50 and XL-75) and was found to increase as the radiation dose increases.

## 4. Discussion

The main purpose of this work was to characterize the wear performance of four different polyethylenes coupled with CoCrMo femoral heads using a 12-stations hip joint simulator for five million cycles. In particular, we asked whether the sole addition of vitamin E on conventional UHMWPE could improve its wear performance in comparison with high cross-linked polyethylene. We observed the highest wear rate on the STD polyethylene cups, followed closely by the VE, whereas the wear rate of XL-50 and XL-75 were sensibly lower than the former, but close to each other. In his research, McKellop et al. [6] found that the process of cross-linking highly improved the wear resistance, and the wear rate decreased markedly with increasing radiation dose, reaching a reduction of the 87% for the cup irradiated at 9.5 Mrad compared to the one irradiated at 3.3 Mrad. Affatato et al. [22] found that the wear of conventional UHMWPE was 40 times higher than for cross-linked polyethylene (XLPE), testing these materials against femoral heads of CoCrMo deliberately scratched.

The adding of vitamin E into the microstructure of conventional polyethylene used for hip components should prevent oxidative degradation and reduce the incidence of fatigue crack, confirming the results obtained by other authors [13]. The clinical consequence of the oxidation is an increased wear rate, starting approximately between 2 and 10 years postoperative [28].

**Figure 7:**
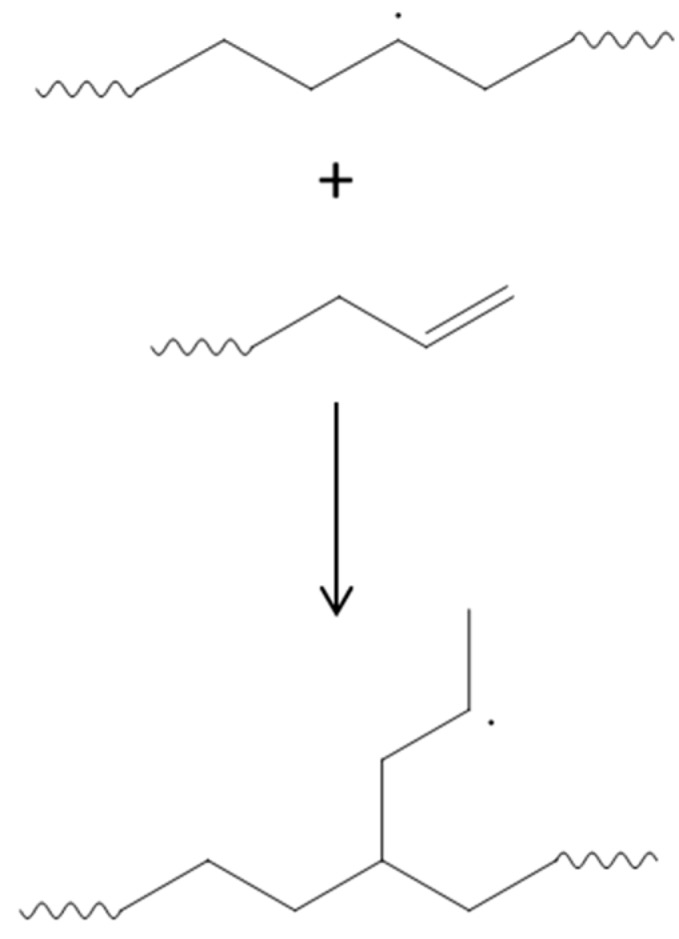
Mechanism of cross-linking of ultra-high-molecular-weight polyethylene (UHMWPE) through consumption of vinyl double bonds.

Trans-Vinylene groups are known to be formed in UHMWPE upon irradiation, and can be used to assess the absorbed radiation dose [29]. The concentration of vinylene double bonds measured in our irradiated samples increases with the irradiation dose, as expected. At the same time, vinyl groups, normally present in virgin UHMWPE, are consumed as a consequence of irradiation, being involved in the formation of Y-shaped cross-links, as shown in Figure 7 [30].

Accordingly, the FTIR analysis of both irradiated samples demonstrates consumption of the vinyl double bonds, while the cross-linking density measurements indicates that those samples have been cross-linked as a consequence of irradiation. Since cross-linking is known to increase the abrasion resistance, this explains the reduced wear, compared to the STD configuration, observed in the wear test. Nevertheless, both XLPE samples showed a significant decrease in crystallinity, and this is known to be correlated with a decrease in fatigue resistance, which creates concerns on their in vivo durability.

On the contrary, VE sample does not show any significant difference in the chemical and physical characteristics, compared to STD UHMWPE, indicating that the addition of vitamin E, which is intended as a stabilizer against oxidation, does not induce any modification to other properties. This also explains the similar behavior observed between VE and STD in the wear test.

From the topographical analysis, a very similar surface pattern of the unworn surfaces of the different polyethylene was found, verifying an almost equal initial condition for all of the cups that underwent the in vitro wear simulation. On the contrary, the inner surface of the worn samples showed large differences, both in terms of qualitative observation and in terms of rigorous measurement of the roughness. The contour images of the cross-linked polyethylene presented a more rough surface, with many signs of delamination and incipient cracks, which are symptoms of the fatigue wear phenomena [31]. On the other side, the non-cross-linked polyethylene presented a smoother surface than the XL ones, which suggests an abrasive wear occurrence that lead to a setting-in phase of the non-cross-linked polyethylene surfaces to the CoCrMo counterbody.

The roughness analysis emphasized these differences in the wear behavior of the polyethylene cups. In fact, higher roughness values on the cross-linked cups than on the standard and vitaminized ones were found. As grooves and scratches are assessed as origin points for surface-initiated micro-cracks, a high roughness value can be an indicator of the fatigue wear mechanism. As expressed in literature, cross-linking affects the compression and tension fatigue of the UHMWPE. Baker et al. [32] found growth of fatigue cracks under fully compressive cyclic loading in notched samples of UHMWPE. Cole et al. [33], in their experimental investigation on the fracture toughness and fatigue-crack resistance of two kinds of molded UHMWPE, found that “gamma irradiation decreased each material’s resistance to fatigue-crack growth, and that the decreases were greater with increased irradiation dose”.

## 5. Conclusions

All the acetabular cups studied in this work showed significant differences with respect to their wear behavior. Lower weight loss was exhibit by the XLPEs, which has potential for reduced wear and decreased osteolysis. The amount of weight loss after five million cycles in a hip joint wear simulator did not correlate with the roughness parameters, while cross-link density is strongly correlated with the wear resistance of UHMWPE. Nevertheless, the sensible higher presence of grooves and scratches on the cross-linked surfaces than on the standard ones is believed to be a plausible cause of fatigue wear. This, with the continuous cyclical compression of the element, could lead to an increased wear rate after elevate amount of working cycles.

Little is known or understood about the relationship between particle debris from PE wear and the biological response leading to osteolysis, and less is known about the role of cross-linking [12,28]. The promise of improved clinical performance in respect to osteolysis is a matter to be determined by long-term (>10 years) clinical follow-up studies. An increased understanding of the related biological processes and osteolysis is needed to provide the ability to better evaluate the STD, XLPEs, and VE performance in vivo.

## Figures and Tables

**Table 1:** Cumulative mass loss (mean ± standard deviation) for the four sets of polyethylene (PE) acetabular cups tested. Values and statistical analysis performed using a Kruskall–Wallis nonparametric test.

Cycles (Mc)	Mean ± Standard Deviation (mg)				K–W Test	Post Hoc Test (*p*-Value)					
	STD	VE	XL-50	XL-75	(*p*-Value)	STD vs. VE	STD vs. XL-50	STD vs. XL-75	VE vs. XL-50	VE vs. XL-75	XL-50 vs. XL-75
0.5	11.4 ± 4.8	10.5 ± 1.2	2.7 ± 2.1	1.7 ± 0.7	0.037	0.821	0.079	0.036	0.047	0.020	0.734
1.0	22.9 ± 3.4	23.3 ± 3.8	9.6 ± 2.8	7.5 ± 1.6	0.035	0.910	0.089	0.024	0.070	0.017	0.571
1.5	27.6 ± 5.2	28.1 ± 3.9	13.0 ± 3.4	10.9 ± 2.3	0.038	0.910	0.070	0.031	0.054	0.024	0.734
2.0	40.9 ± 7.3	39.0 ± 5.5	17.4 ± 2.7	13.9 ± 2.4	0.030	0.910	0.089	0.013	0.113	0.017	0.428
2.5	54.1 ± 5.0	60.13 ± 8.8	22.5 ± 4.3	17.2 ± 2.4	0.026	0.571	0.174	0.031	0.054	0.007	0.428
3.0	67.0 ± 10.5	65.2 ± 9.1	25.7 ± 4.6	21.0 ± 1.9	0.035	0.910	0.070	0.017	0.089	0.024	0.571
3.5	80.8 ± 12.2	73.7 ± 14.2	31.2 ± 4.5	24.3 ± 2.6	0.030	0.910	0.089	0.013	0.113	0.017	0.428
4.0	89.4 ± 11.3	82.4 ± 13.3	34.6 ± 4.9	26.6 ± 2.8	0.030	0.910	0.089	0.013	0.113	0.017	0.428
4.5	94.8 ± 10.0	88.1 ± 13.6	37.3 ± 5.1	29.3 ± 2.7	0.030	0.910	0.089	0.013	0.113	0.017	0.428
5.0	102.6 ± 15.1	94.9 ± 16.7	39.3 ± 6.7	31.8 ± 2.6	0.032	0.910	0.079	0.015	0.100	0.020	0.496

Statistical significance: *p* < 0.05.

**Table 2:** Physical–chemical characteristics of the control samples, resulting from FTIR, DSC, and cross-link density measurements.

Polyethylene	Crystallinity (%)	Trans-Vinylene (mmol/L)	Cross-Link Density (mol/dm^3^)
STD	50.3 ± 0.9	-	-
VE	51.2 ± 1.2	-	-
XL-50	40.8 ± 1.0	5.2	0.132 ± 0.009
XL-75	35.5 ± 0.9	5.8	0.139 ± 0.010

## Supplementary Materials

The following are available online at http://www.mdpi.com/1996-1944/11/3/433/s1, Figure S1: Supplementary figure with a second set of contour images of the topographies acquired on the worn inner surfaces of four loaded specimens: a VE; b STD; c XL-75; d XL-50. The observations are very similar to the ones described for Figure 4.
