# Supplementary material for: Wear Behaviours and Oxidation Effects on Different UHMWPE Acetabular Cups Using a Hip Joint Simulator
Source: Materials (Basel). 2018 Mar 16;11(3):433. doi: 10.3390/ma11030433 (PMC5873012; doi:10.3390/ma11030433)
Supplement: Supplementary file 1 [file materials-11-00433-s001.pdf]

Supplementary

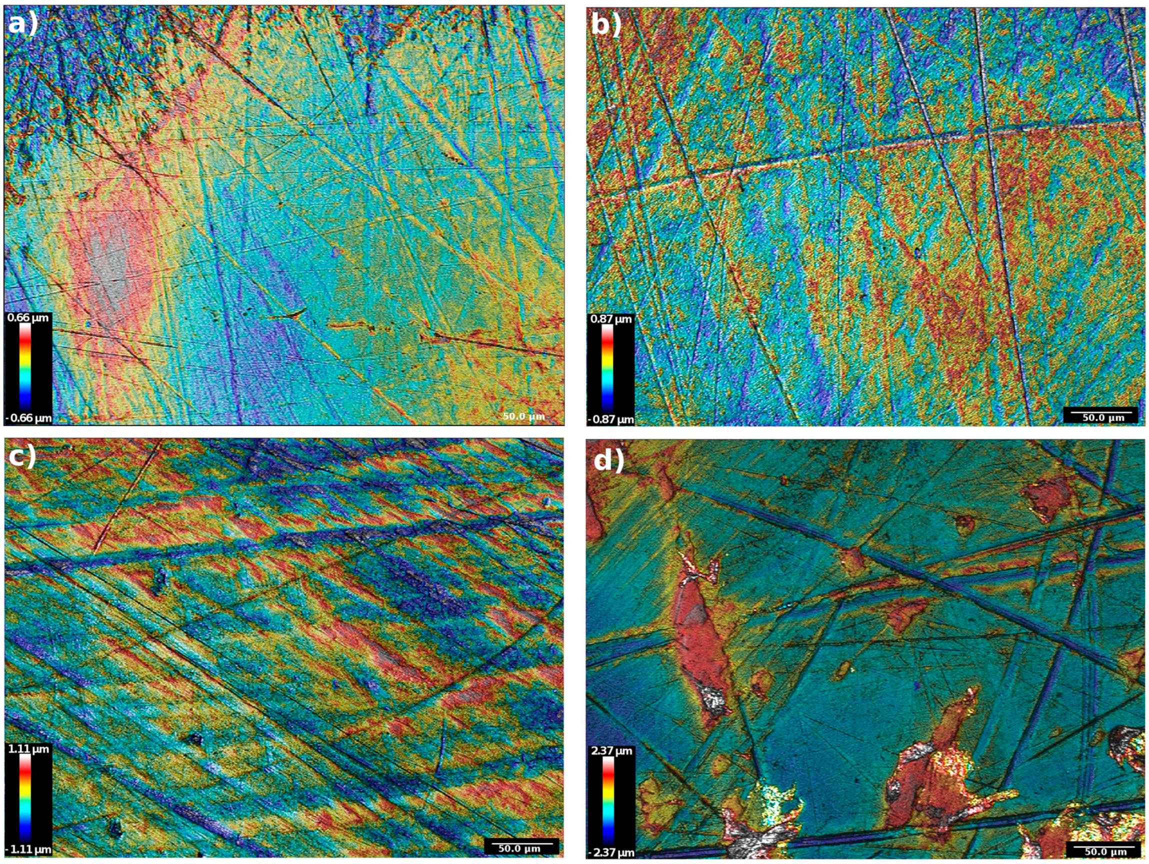

Figure S1 – *Supplementary figure with a second set of contour images of the topographies acquired on the worn inner surfaces of four loaded specimens: a VE; b STD; c XL-75; d XL-50. The observations are very similar to the ones described for Figure 4.*
